# LGR5 in breast cancer and ductal carcinoma in situ: a diagnostic and prognostic biomarker and a therapeutic target

**DOI:** 10.1186/s12885-020-06986-z

**Published:** 2020-06-10

**Authors:** Catharina Hagerling, Mark Owyong, Vaishnavi Sitarama, Chih-Yang Wang, Charlene Lin, Renske J. E. van den Bijgaart, Charlotte D. Koopman, Audrey Brenot, Ankitha Nanjaraj, Fredrik Wärnberg, Karin Jirström, Ophir D. Klein, Zena Werb, Vicki Plaks

**Affiliations:** 1 Department of Anatomy and the Helen Diller Family Comprehensive Cancer Center, University of California, San Francisco, CA 94143-0452 USA; 2 Department of Clinical Sciences Lund, Division of Oncology and Pathology, Lund University, SE-221 85 Lund, Sweden; 3 Present Address: Department of Laboratory Medicine, Division of Clinical Genetics, Lund University, SE-221 85 Lund, Sweden; 4 Department of Biochemistry and Molecular Biology, Institute of Basic Medical Sciences, College of Medicine, National Cheng Kung University, Tainan, Taiwan; 5 Present Address: Radiotherapy and Oncoimmunology Laboratory, Department of Radiation Oncology, Radboud University Medical Center, Geert Grooteplein Zuid 32, 6525 GA Nijmegen, Netherlands; 6 Present Address: Department of Medical Physiology, Division of Heart & Lungs, University Medical Center Utrecht, Yalelaan 50, 3584CM Utrecht, Netherlands; 7 Hubrecht Institute, Royal Netherlands Academy of Arts and Sciences (KNAW), University Medical Centre Utrecht, 3584CT Utrecht, Netherlands; 8 Present Address: ICCE Institute, School of Medicine, Department of Medicine, Washington University, St Louis, MO 63110 USA; 9 Department of Surgery, Institute of Clinical Sciences, Sahlgrenska Academy at the University of Gothenburg, Sahlgrenska University Hospital, S413 45 Gothenburg, Sweden; 10 Department of Orofacial Sciences, University of California, 513 Parnassus Avenue, San Francisco, CA 94143-0452 USA; 11 Institute for Human Genetics, University of California, San Francisco, San Francisco, CA 94143 USA

**Keywords:** LGR5, Breast cancer, DCIS, Estrogen receptor, Targeted therapy

## Abstract

**Background:**

Novel biomarkers are required to discern between breast tumors that should be targeted for treatment from those that would never become clinically apparent and/or life threatening for patients. Moreover, therapeutics that specifically target breast cancer (BC) cells with tumor-initiating capacity to prevent recurrence are an unmet need. We investigated the clinical importance of LGR5 in BC and ductal carcinoma in situ (DCIS) to explore LGR5 as a biomarker and a therapeutic target.

**Methods:**

We stained BC (*n =* 401) and DCIS (*n* = 119) tissue microarrays with an antibody against LGR5. We examined an *LGR5* knockdown ER^−^ cell line that was orthotopically transplanted and used for in vitro colony assays. We also determined the tumor-initiating role of Lgr5 in lineage-tracing experiments. Lastly, we transplanted ER^−^ patient-derived xenografts into mice that were subsequently treated with a LGR5 antibody drug conjugate (anti-LGR5-ADC).

**Results:**

LGR5 expression correlated with small tumor size, lower grade, lymph node negativity, and ER-positivity. ER^+^ patients with LGR5^high^ tumors rarely had recurrence, while high-grade ER^−^ patients with LGR5^high^ expression recurred and died due to BC more often. Intriguingly, all the DCIS patients who later died of BC had LGR5-positive tumors. Colony assays and xenograft experiments substantiated a role for LGR5 in ER^−^ tumor initiation and subsequent growth, which was further validated by lineage-tracing experiments in ER^−^ /triple-negative BC mouse models. Importantly, by utilizing LGR5^high^ patient-derived xenografts, we showed that anti-LGR5-ADC should be considered as a therapeutic for high-grade ER^−^ BC.

**Conclusion:**

LGR5 has distinct roles in ER^−^ vs. ER^+^ BC with potential clinical applicability as a biomarker to identify patients in need of therapy and could serve as a therapeutic target for high-grade ER^−^ BC.

## Background

Overtreatment is a major challenge in the clinical management of breast cancer [1, 2]. Two patient groups subjected to overtreatment are those with ductal carcinoma in situ (DCIS) and those with estrogen receptor-positive (ER^+^) breast cancer (BC) [3, 4].

Gene expression profiling of BC has enabled its classification into four clinically relevant BC subtypes; two ER^+^ subtypes (Luminal A and Luminal B) with favorable clinical outcomes as compared to the two ER^−^ subtypes (triple negative/basal-like and human epidermal growth factor receptor 2 (HER2) enriched) [5–8]. Patients with low-grade, < 2 cm, ER^+^ tumors have the most favorable outcome. DCIS tumors are a heterogeneous group of pre-invasive lesions [9–11]. While some DCIS tumors are predestined to acquire an invasive phenotype and progress into bona fide BC, others never become invasive [9]. It is known that approximately 25–50% of DCIS tumors progress into invasive BC independent of DCIS tumor grade [12–15], a finding supported by genomic and transcriptomic data showing that DCIS share genetic similarities with invasive BC [16–19]. Similar to patients with DCIS tumors, approximately 50% of patients with low-grade ER+ tumors might end up receiving therapy without deriving clinical benefit [20]. Thus, to optimize patient care, there is a need to uncover novel diagnostic and prognostic biomarkers to discern between tumors that never become invasive and/or life threatening from those that should be targeted for treatment.

Targeted therapies against hormone receptors and HER2 in BC have improved overall survival over the past two decades, with the 5-year survival rates in several countries now approaching 90% [21]. Despite these advances in risk stratification and therapy, treatment of patients with triple-negative BC (TNBC) remains a major clinical challenge. TNBC, an ER^−^, progesterone receptor (PR) negative and HER2^−^ BC, is more likely to recur and disseminate with a lower 5-year survival rate as compared to other BC subtypes [22]. About 70–80% of these cancers are also basal-like since they bear characteristics of basal epithelial breast cells [23–25]. The prognosis of TNBC tumors is often poor with limited effective treatment options [23, 24]. Additionally, many TNBC patients have metastatic disease at the time of diagnosis that responds poorly to conventional treatments, such as chemo- and radiotherapy [26]. Apart from a recent clinical study conducted by Genentech using chemotherapy, paclitaxel, and anti-PD-L1 checkpoint inhibitor, atezolizumab, that showed 40% greater progression free survival as compared to chemotherapy alone [27], there are currently no other approved targeted therapies for TNBC.

To uncover novel diagnostic and prognostic BC biomarkers that could potentially provide a backbone for the future development of targeted therapeutics, we interrogated the leucine-rich repeat containing G-protein-coupled receptor 5 (LGR5). LGR5 potentiates canonical Wnt signaling and marks stem cells in various tissues, including the mammary gland [28–32]. We and others have previously shown that Lgr5 identifies bipotent mammary stem cells (MaSC) that are able to differentiate into the two primary epithelial cell lineages of normal mammary glands, i.e., luminal and myoepithelial cells [33–35]. Importantly, accumulating data indicate that Lgr5 marks cancer cells with tumor initiating capacity [36–38], which possess characteristics of cancer stem cells (CSCs). CSCs are tumor cells that can self-renew and have clonal tumor initiating capacity alongside long-term repopulation potential to facilitate recurrence and metastasis. Experimental models and clinical studies have indicated that CSCs survive many conventional cancer therapeutics [39].

We hypothesize that LGR5, an established CSCs marker in several cancers including BC [40], can be used to identify at-risk BC patients that would benefit from treatment and can also serve as a therapeutic target for high-grade ER^−^ BC lesions.

## Methods

### DCIS TMA and BC TMA

The BC tissue microarray (TMA) was generated from a consecutive series of tumors from patients diagnosed with primary invasive BC at the Department of Pathology, Malmö University Hospital between 1988 and 1992 with a median follow-up of 106 months. The cohort is described in detail elsewhere [41]. ER^+^ was determined as over 10% positive cells, according to national guidelines. Of all 498 cases originally included in the TMA, only 401 could be annotated for LGR5 due to tumor loss or low tumor cell content. The mean and median age at diagnosis was 65 years, ranging from 28 to 96 years of age. Ethical permits were obtained from the ethical committee at Lund University.

The DCIS TMA included women from a population-based cohort diagnosed with primary DCIS between 1986 and 2004 in two different Swedish counties and followed until November 2011. The cohort is described in detail elsewhere [42]. Due to either tumor loss or low tumor cell content, only 119 DCIS tumors (originally 480 cases) were annotated for LGR5. The mean age at diagnosis for these patients were 57 years, ranging from 30 to 90 years of age, with a median age of 58 years. ER^+^ was determined as over 10% positive tumor cells, according to national guidelines. Ethical permits were obtained from the ethical committee at Uppsala University Hospital.

### Immunohistochemistry and annotation

TMA sections mounted on glass slides were deparaffinized prior to antigen retrieval and stained with anti-LGR5 antibody (Abcam ab75732). The staining intensity of LGR5 was evaluated in tumor cells and scored from 0 to 3 with 0–1 denoting low and 2–3 denoting high. The staining intensity was evaluated by C. H and C. L (Cohen’s kappa, 0.41), blinded to all clinical information during scoring, first separately then interpretation combined.

### Cell lines

For generation of the *MDA-MB-231 LGR5 KD* (MDA-LGR5KD) cell line viral production was carried out using TransIT-LT1 (Mirus Bio) mediated transfection of HEK293T cells. Virus was added to the cells with Polybrene and MDA-MB-231 (MDA-ctrl), a TNBC cell line, was transduced with pLKO.1-TRC containing shSCR or shLgr5 sequences [43]. Stably transduced cells were selected in puromycin for at least 5 days. Knockdown of *Lgr5* was confirmed by qPCR. The MCF7 cell line, a Luminal A BC cell line, was used to compare TNBC to luminal BC. All cell lines were cultured in DMEM high-glucose + 10% fetal bovine serum + 1% Penicillin Streptomycin. Cells were tested for Mycoplasma by PCR amplification using primers Myco+ (5′-GGG AGC AAA CAG GAT TAG ATA CCC T-3′) and Myco- (5′-TGC ACC ATC TGT CAC TCT GTT AAC CTC-3′) every 6 months and treated for a minimum of 2 weeks with Plasmocin (InvivoGen) if the Mycoplasma PCR was positive, until the PCR was negative.

### RNA extraction and real-time PCR

Total RNA was extracted from the MDA-ctrl, MDA-LGR5KD, and MCF7 cell lines using the RNeasy kit (Qiagen). For total RNA extraction from wild-type, C3(1)-Tag, and MMTV-PyMT mammary glands/tumors, tumor bearing mice were staged according to well-known time windows of hyperplasia, adenoma, and carcinoma [44, 45]. Mammary glands were surgically extracted, flash frozen, and pulverized. RNA extraction was performed using RNeasy kit. Reverse transcription was performed using iScript from Biorad. RNA quantity was analyzed with a NanoDrop spectrophotometer. Real-time PCR (rtPCR) was performed in a RealPlex2 (Eppendorf). Data was normalized to GAPDH for both human and mouse rtPCR analyses.

**Table:** 

**RT-PCR Primer List - Human**		
**Gene**	**Forward Primer (5′ -- > 3′)**	**Reverse Primer (5′ -- > 3′)**
Lgr5	TCTTCACCTCCTACCTGGACCT	GGCGTAGTCTGCTATGTGGTGT
Cyclin D	ATGTTCGTGGCCTCTAAGATGA	CAGGTTCCACTTGAGCTTGTTC
c-Myc	AAAGGCCCCCAAGGTAGTTA	GCACAAGAGTTCCGTAGCTG
GAPDH	AACGGGAAGCCCATCACCATCTT	CAGCCTTGGCAGCACCAGTGG
**RT-PCR Primer List - Mouse**		
**Gene**	**Forward Primer (5′ -- > 3′)**	**Reverse Primer (5′ -- > 3′)**
Lgr4	CCCGACTTCGCATTCACCAA	GCCTGAGGAAATTCATCCAAGTT
Lgr5	ACATTCCCAAGGGAGCGTTC	ATGTGGTTGGCATCTAGGCG
Lgr6	ATCATGCTGTCCGCTGACTG	ACTGAGGTCTAGGTAAGCCGT
GAPDH	TGCACCACCAACTGCTTAG	GGATGCAGGGATGATGTTC

### 3D colony forming assay

3000 cells of MDA-ctrl or MDA-LGR5KD were seeded in 50 μl of 1:1 Matrigel:medium (Gibco DMEM high-glucose, 10% fetal bovine serum, 1% Penicillin Streptomycin) and plated onto 6-well plates. Cells were monitored for spheroid formation on days 1, 4 and 6.

### Cleared mammary fat pad transplantation of MDA-MB-231 cell lines

For mammary orthotopic transplants, 10^6^ tumor cells plus 10^5^ human cancer-associated fibroblasts (previously isolated [46] and immortalized utilizing insertion of hTERT into patient-derived fibroblasts [47, 48]) were injected in the right inguinal fat pad number 4 of SCID/NCr mice on the BALB/c background (Charles River) in 50 μl Matrigel. Tumor diameter was measured with calipers every week for 6 weeks. All procedures involving animals and their care were performed in accordance with the guidelines of the American Association for Accreditation for Laboratory Animal Care and the U.S. Public Health Service Policy on Human Care and Use of Laboratory Animals. All animal studies involving fat pad transplantation of MDA-MB-231 cell lines were approved and supervised by the Washington University Institutional Animal Care and Use Committee in accordance with the Animal Welfare Act, the Guide for the Care and Use of Laboratory Animals and NIH guidelines (Protocol 20,150,145).

### Cleared mammary fat pad transplantation of PDX and treatment with antibodies

Patient derived xenograft (PDX) tumors were implanted as ~8mm^3^ fragments into the right inguinal fat pad of NOD/SCID mice between 4 and 6 weeks of age following established protocols [49]. Mice were monitored until the tumors reached 150–200 mm^3^ then intra-peritoneally injected with anti-LGR5-vc-MMAE antibody drug conjugate (ADC) [12 mg/kg] once/week for 4 weeks. Control mice were treated with anti-gD-MC-vc-PAB-MMAE (12 mg/kg). Both control and experimental mice were also treated with gD-5237 (30 mg/kg), a non-binding antibody control, 4 h prior to treatment with anti-LGR5 ADC. Mice were dissected 4 days following the last treatment. All animal experiments involving fat transplantation of PDX were reviewed and approved by the Institutional Animal Care and Use Committee (IACUC) at the University of California, San Francisco.

### Lineage tracing

Lgr5-EGFP-IRES-creERT2/Rosa-Tomato/C3-Tag (1) mice were generated by crossing Lgr5-EGFP-IRES-creERT2/Rosa-Tomato C57Bl/6 mice [36] with C3-Tag (1) FVB mice [50]. 7-week-old Lgr5-EGFP-IRES-creERT2/Rosa-Tomato/C3-Tag (1) mice were treated with 5 mg tamoxifen (Sigma cat#T5648-1G) in 200 ul sunflower seed oil (Sigma cat#S5007-250ML) 3 times/week for 1 week. Hyperplastic mammary glands were harvested and imaged for fluorescence on the Leica MZ16F stereoscope, 4 days following the end of treatment. All animal experiments involving lineage tracing were reviewed and approved by the Institutional Animal Care and Use Committee (IACUC) at the University of California, San Francisco.

### Bioinformatics and computational analysis of high-throughput data

The clinical and gene expression data of BC from the METABRIC database were deposited in European Genotype Archive (http://www.ebi.ac.uk/ega/page.php according to the accession number: EGAS00000000083 and can be downloaded from the Oncomine platform (www.oncomine.org) [51]. Statistical analysis of the differences in LGR5 transcript expression in different tissues were calculated by Oncomine standard algorithms: for each microarray, data were log2-transformed, median-centered, and normalized with standard deviation.

### Statistics

GraphPad Prism7 was used to perform the statistical analysis. *P* values were generated using the unpaired Student’s t-test between two groups, whereas a one-way analysis of variance was employed to determine two or more group differences with Tukey’s multiple comparisons. *P*-values ≤0.05 were considered significant.

Spearman’s Rho tests were used for correlation analysis and Kaplan-Meier analysis with log-rank test was used to depict differences in recurrence and death due to BC. Cox regression proportional hazards models were used for estimation of hazard ratios (HR) for recurrence and for death from breast cancer in both uni- and multivariable analysis. Covariates with a *P* value < 0.05 in the univariable analysis were included in the multivariable analysis. All statistical tests were two sided and *P* value ≤0.05 was considered significant. IBM SPSS Statistics version 24 was used to perform the analysis.

The remark criteria were used to report the data [52].

## Results

### Clinical importance of LGR5 in BC and its prognostic value

**Fig. 1:**
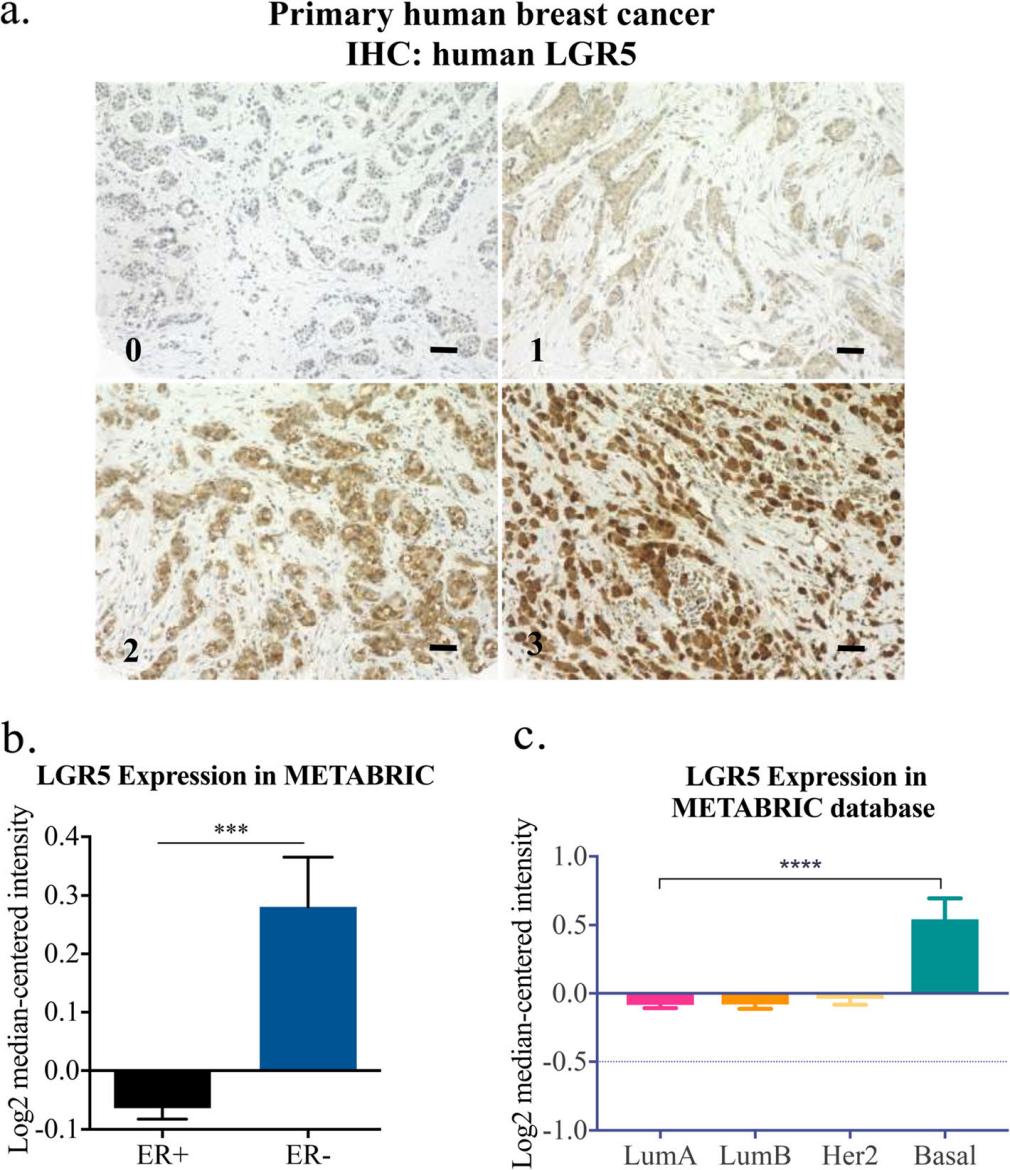
LGR5 and clinicopathological features in BC. **a** Representative images of LGR5-stained primary human BC (numbers on bottom left of each image indicate score for tumor staining intensity). Scale bar, 50 μM. **b** Analysis of patient samples in METABRIC database for *LGR5* expression in >ER^−^ vs. ER^+^ BC show elevated *LGR5* expression in ER^−^ BC. **c** Various BC subtypes with Basal BC showing a statistically significant elevation in *LGR5* expression compared to Luminal A, Luminal B, and Her2, mean ± SEM. (Student *t-*test, ***P < 0.001, ****P < 0.0001)

To decipher the role of LGR5 in BC we stained a BC TMA (Fig. 1a). LGR5 negatively correlated to tumor size (*P =* 0.001), nodal metastasis (*P =* 0.002), Nottingham histological grade (NHG) (*P =* 0.047) and ER (*P =* 0.001) (Table 1). Further investigation of BC patients included in the METABRIC database was in line with our data showing that LGR5 gene expression was significantly higher in ER^−^ BC and basal subtypes (Fig. 1b-c).

**Table 1:** Correlation between LGR5 expression and clinicopathologic features in primary breast cancer

Variable	Patients N (%)	LGR5 intensity				R	***P*** value
		0	1	2	3		
All	401 (100)	69	157	151	24		
Age							
< 50	67 (16.7)	8	32	23	4	- 0.003	0.95
≥ 50	334 (83.3(	61	125	128	20		
Tumor size							
≤ 20 mm	232 (57.9)	32	82	104	14	−0.162	**0.001**
> 20 mm	169 (42.1)	37	75	47	10		
Ki67							
0–10%	139 (36.4)	27	48	57	7	0.012	0.81
11–25%	127 (33.2)	23	45	43	9		
> 25%	116 (30.4)	17	48	43	8		
NHG							
1	93 (23.3)	13	31	44	5	−0.100	**0.047**
2	161 (40.3)	30	58	63	10		
3	146 (36.5)	26	67	44	9		
Nodal metastasis							
No	208 (57.8)	24	86	87	11	−0.162	**0.002**
Yes	152 (42.2)	38	60	49	5		
ER							
≤ 10%	61 (15.2)	3	23	26	9	−0.166	**0.001**
> 10%	340 (84.4)	66	134	125	15		
PR							
≤ 10%	125 (31.2)	20	45	48	12	−0.70	0.16
> 10%	276 (68.8)	49	112	103	12		
Luminal A	214 (53.6)	41	87	77	9	−0.092	0.065
Luminal B	35 (8.8)	8	13	13	1	−0.043	0.40
HER2	22 (5.5)	0	9	10	3	0.110	**0.028**
Basal	136 (34.3)	15	55	55	11	0.103	**0.040**

*Abbreviations*: *LGR5* Leucine-rich repeat-containing G-protein coupled receptor 5, *R* correlation coefficient, *NHG* Nottingham histologic score, *ER* estrogen receptor, *PR* progesterone receptor, *HER2* human epidermal growth factor receptor. Spearman correlation, two-tailed- value. Bold indicates *P* -value < 0.05

**Fig. 2:**
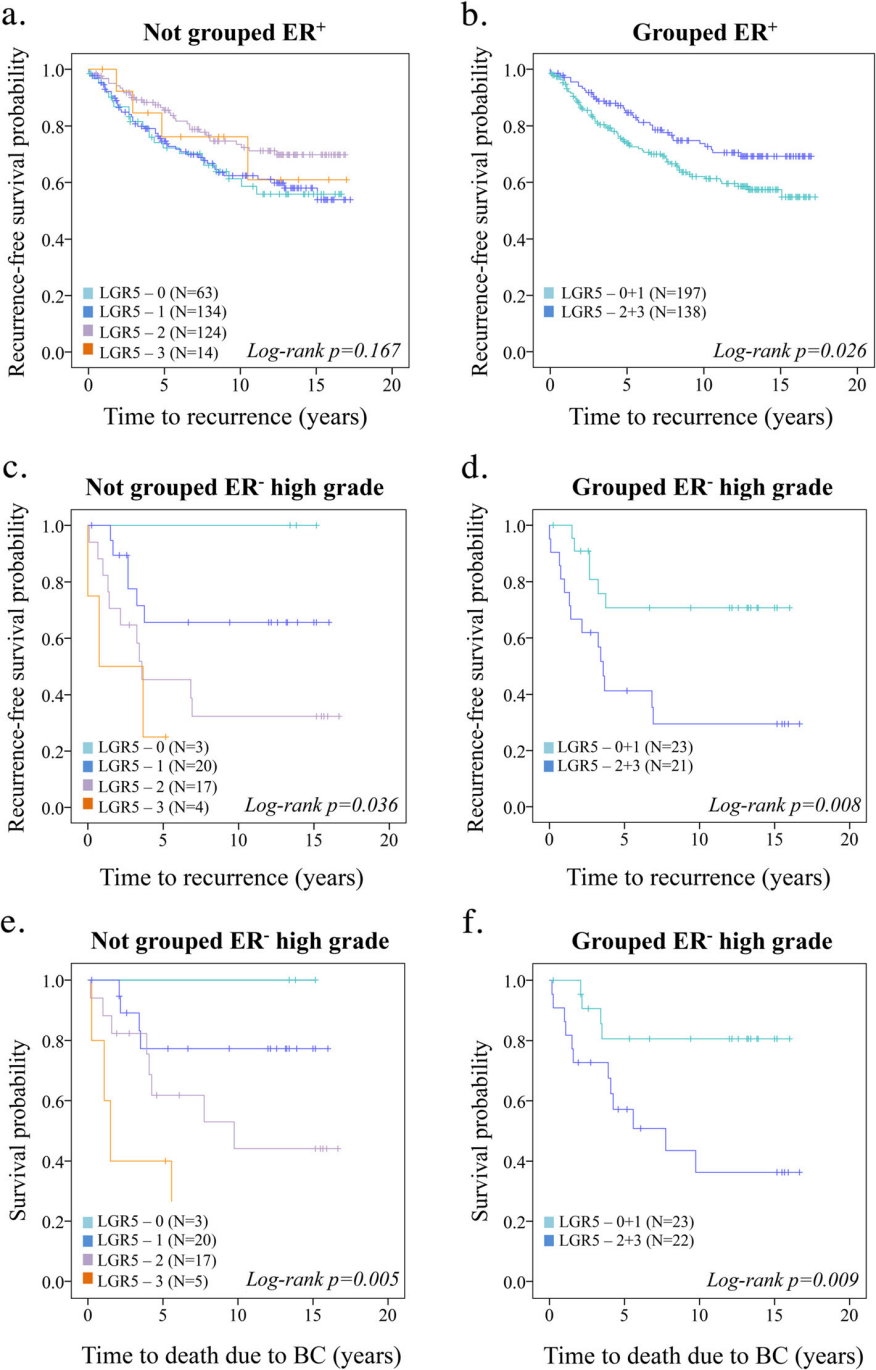
Prognostic impact of LGR5 in ER^+^ vs. ER^−^ BC. **a** Kaplan-Meier plots (log-rank test) displaying recurrence-free survival according to LGR5 in ER^+^ BC not-grouped and **b** LGR5 dichotomized into LGR5^low (0–1)^ and LGR5^int/high (2–3)^ tumor staining intensity. **c** Kaplan-Meier plots (log-rank test) displaying recurrence-free survival according to LGR5 in high-grade ER^−^ BC not-grouped and **d** LGR5 dichotomized into LGR5^low (0–1)^ and LGR5^int/high (2–3)^ tumor staining intensity. **e** Kaplan-Meier plots (log-rank test) displaying BC-specific survival according to LGR5 in high-grade ER^−^ BC not-grouped or **f** Dichotomized into LGR5^low (0–1)^ and LGR5^int/high (2–3)^ tumor staining intensity

Next, we evaluated the prognostic role of LGR5 for recurrence-free survival (RFS) among BC patients. Since we did not observe any overall prognostic significance of LGR5 in BC patients (Additional Fig. 1a-b), we stratified the analysis for ER^−^ and ER^+^ tumors. A total of 337 patients (85%) had ER^+^ tumors. LGR5 staining intensities were dichotomized into LGR5^low(0 + 1)^ and LGR5^high(2 + 3)^. LGR5^high^ patients accounted for 41.3% of all ER^+^ BC patients and 36% (*N* = 37) of these patients experienced recurrence during follow-up, whereas 56% (*N* = 71) of the patients with LGR5^low^ tumors recurred (*P = 0.024;* Fig. 2a-b). Univariable cox regression analysis showed that ER^+^LGR5^high^ BC tumors had a statistically significant decrease in recurrence rate [*P =* 0.025, HR 0.632 (95% CI, 0.42–0.95), (Additional Table 1)]. However, LGR5 was not an independent prognostic factor as determined with multivariable cox regression analysis (Additional Table 1).

We then evaluated the prognostic value of LGR5 in ER^−^ BC. We found that LGR5 had a prognostic significance for high-grade ER^−^ BC patients only (Fig. 2c-f and Additional Fig. 1c-d). High-grade ER^−^ BC patients with LGR5^high^ tumors more frequently experienced recurrence (*P =* 0.008) and death due to BC (*P =* 0.009). Univariable cox regression analysis showed that high-grade ER^−^LGR5^high^ BC tumors had a statistically significant increase in recurrence rate [*P = 0.013*, HR 3.36 (95% CI, 1.29–8.77)] (Additional Table 2) and death due to BC [*P = 0.016*, HR 4.03 (95% CI, 1.29–12.55)] (Additional Table 3). LGR5 was a borderline independent prognostic factor as determined with multivariable cox regression analysis for recurrence [*P = 0.065*, HR 2.64 (95% CI, 0.94–7.43)] (Additional Table 2) and death due to BC [*P = 0.075*, HR 3.08 (95% CI, 0.89–10.60)] (Additional Table 3).

### Clinical importance of LGR5 in DCIS and its prognostic value

**Fig. 3:**
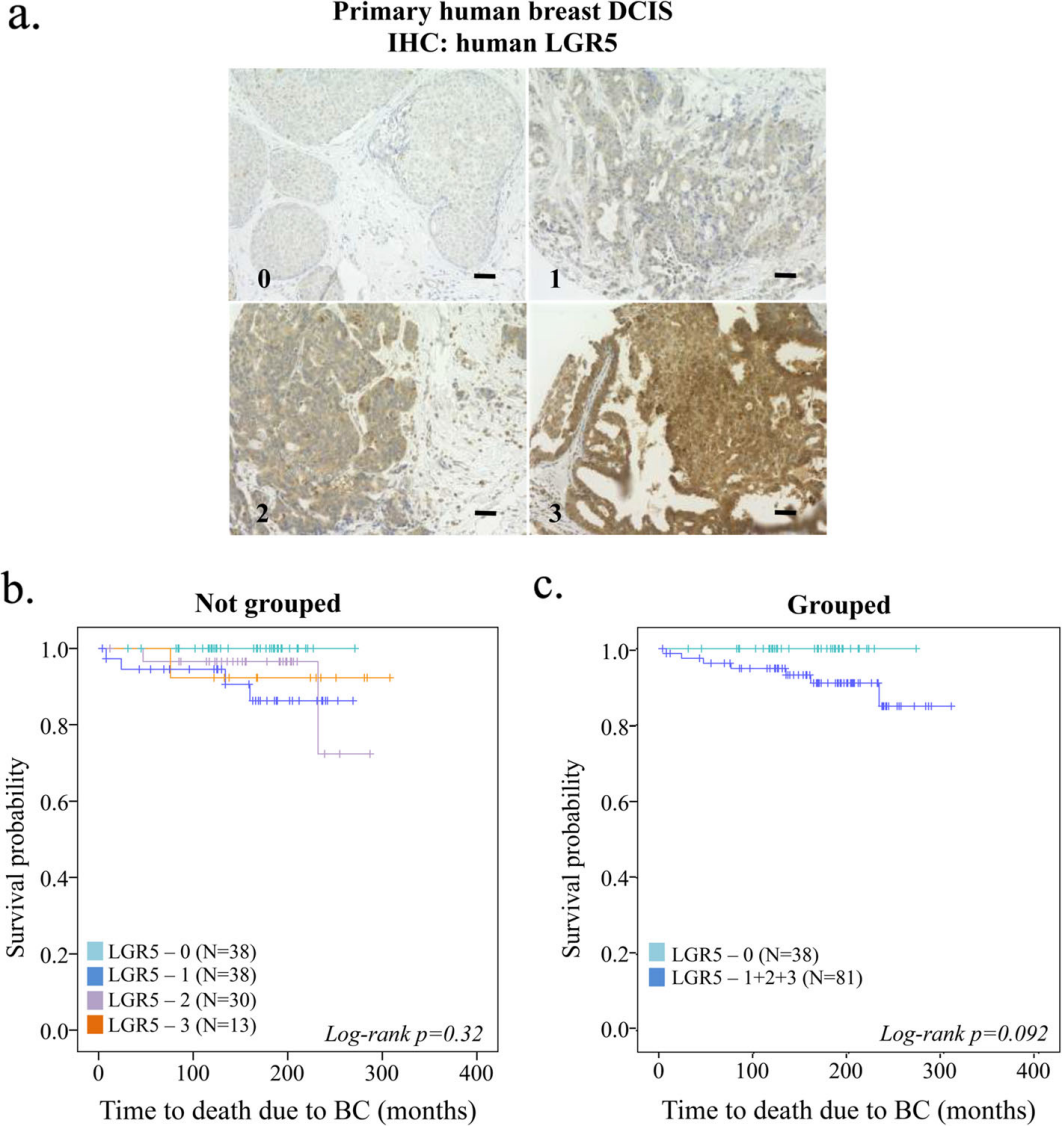
Clinical importance of LGR5 in DCIS and its prognostic value. **a** Representative images of LGR5-stained primary human DCIS tumors (numbers on bottom left of each image indicate score for tumor staining intensity). Scale bar, 50 μM. **b** Kaplan-Meier plots (log-rank test) displaying time to death due to BC according to LGR5 staining intensity not-grouped or **c** LGR5 dichotomized into LGR5^low (0)^ and LGR5^int/high (1 + 2 + 3)^ tumor staining intensity

To further explore the importance of LGR5 in BC initiation and its clinical importance, we analyzed correlations between various clinicopathological features and LGR5 protein expression in DCIS (Fig. 3a). While there was no significant correlation to age, proliferation, nuclear grade, ER or PR, LGR5 expression correlated with tumor size [*P =* 0.01] (Table 2).

**Table 2:** Correlation between LGR5 expression and clinicopathologic features in DCIS

Variable	Patients N (%)	LGR5 intensity				R	***P*** value
		0	1	2	3		
All	119 (100)	38	38	30	13		
Age							
< 50	38 (31.9)	13	11	11	3	0.027	0.77
≥ 50	81 (68.1)	25	27	19	10		
Tumor size							
≤ 20 mm	73 (76)	29	24	14	6	0.26	**0.01**
> 20 mm	23 (24)	3	8	9	3		
Proliferation							
< 10%	60 (57.1)	19	15	16	10	−0.046	0.63
≥ 10%	45 (42.9)	10	20	13	2		
Nuclear grade							
1	11 (9.2)	6	3	1	1	0.081	0.38
2	46 (38.7)	14	14	13	5		
3	62 (52.1)	18	21	16	7		
ER							
≤ 10%	33 (29.5)	8	9	11	5	−0.163	0.085
> 10%	79 (70.5)	28	27	17	7		
PR							
≤ 10%	52 (46.8)	15	15	16	6	−0.086	0.37
> 10%	59 (53.2)	20	20	13	6		

*Abbreviations*: *LGR5* Leucine-rich repeat-containing G-protein coupled receptor 5, *R* correlation coefficient, *ER* estrogen receptor, *PR* progesterone receptor; Proliferation by IH or S-phase Spearman correlation, two-tailed *P* -value. Bold indicates *P* -value < 0.05

Among the 119 patients for whom LGR5 staining was performed, 7 women died due to BC during the 169 months follow-up. All of the women who later died of BC had LGR5-positive (LGR5^int/high(1–3)^) DCIS tumors, while none of the women with LGR5^low(0)^ experienced BC-related death, accounting for 32% of all DCIS patients (Fig. 3b-c). The potential of LGR5 as a prognostic biomarker appeared to be independent of the ER status of the DCIS tumors (Additional Fig. 2a-b). Of note, there was no major difference in the distribution of in situ or invasive relapse between LGR5^0^ and LGR5^1–3^ DCIS tumors (Additional Fig. 2c).

### The tumorigenic role of LGR5 in ER^−^ BC

**Fig. 4:**
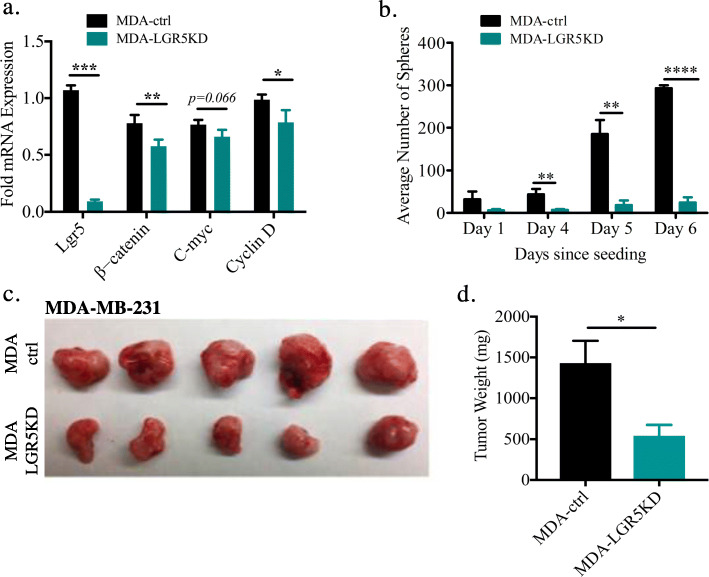
The tumorigenic role of LGR5 in ER^−^ BC. **a** Gene expression profiles in control and LGR5-KD MDA-MB-231 (MDA) cell lines. KD of LGR5 downregulates canonical Wnt pathway genes, such as β*-catenin* and *Cyclin D* (*n* = 3, Mean ± SD, Student *t-*test, **P < 0.01, ***P < 0.001). **b** Colony assay as a surrogate for tumor initiating capacity using single MDA-ctrl and MDA-LGR5KD cells in 3D culture showing number of spheres across increasing days in culture. KD of LGR5 significantly reduces sphere formation when compared to MDA-ctrl (*n* = 3, Mean ± SD, Student *t-*test, *P < 0.05, **P < 0.01, ****P < 0.0001). **c** Representative images from resected tumors of 1 × 10^6^ MDA cells transplanted in Matrigel that were allowed to grow in the inguinal mammary gland for 6 weeks. **d** Weights of resected tumors from MDA-ctrl (*n* = 4) and MDA-LGR5KD cell lines (*n* = 4) shows that KD of LGR5 attenuates tumor growth. Mean ± SD. (Student *t-*test, *P < 0.05)

To further assess the importance of LGR5 in high-grade ER^−^ BC, and specifically, whether LGR5 could serve as a therapeutic target for this particular patient group, we turned to in vitro and in vivo studies. Using the ER^−^ MDA-MB-231 (MDA-ctrl) cell line, a TNBC cell line, to knockdown (KD) *LGR5* expression with siRNA (MDA-LGR5KD), we noticed a decrease in LGR5-associated genes, e.g., genes part of the canonical Wnt pathway [β-catenin and cyclin D] (Fig. 4a, Additional Fig. 3a). The association of LGR5 with Wnt pathway genes, in particular β-catenin, was previously shown to be important in TNBC utilizing the MDA-MB-231 and MDA-MB-453 cell lines [53, 54]. To investigate the importance of LGR5 in colony-formation that evaluates tumor-initiating capacity, we performed an in vitro sphere assay using MDA-ctrl and MDA-LGR5KD cells. MDA-LGR5KD cells exhibited significant reduction in their ability to form spheres (Fig. 4b). In vivo studies with these cells further confirmed that MDA-LGR5KD cells form smaller tumors as measured by both size and weight (Fig. 4c-d). These data are in line with a previous publication [37].

### Targeting LGR5+ cells in ER^−^ BC due to their tumor-initiating capacity

**Fig. 5:**
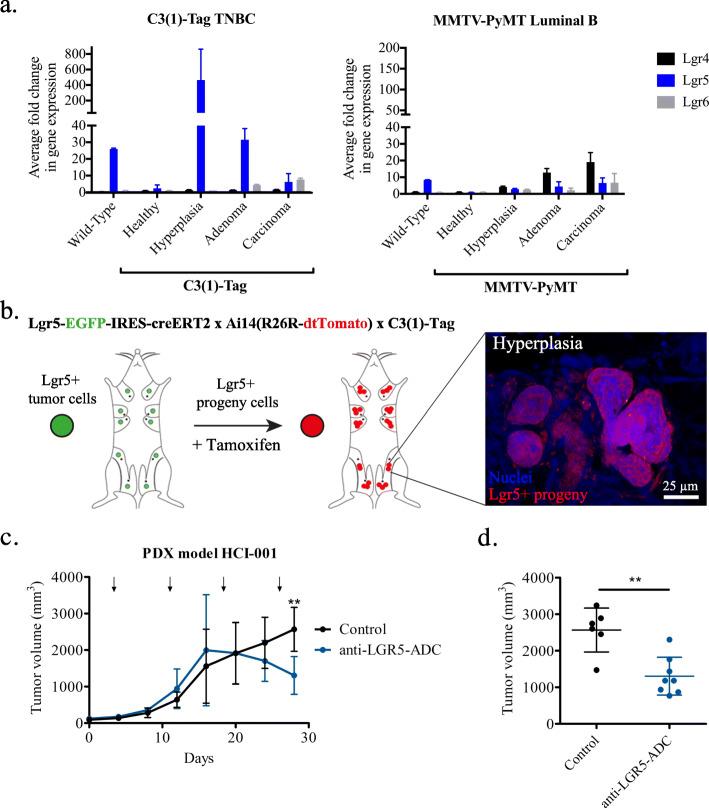
Targeting LGR5+ cells in ER^−^ BC due to their tumor-initiating capacity. **a** Quantitative polymerase chain reaction for gene expression analysis of *Lgr4*, *Lgr5*, and *Lgr6* in the C3(1)-Tag TNBC spontaneous BC mouse model and MMTV-PyMT Luminal B spontaneous BC mouse model. C3(1)-Tag mice have a greater fold-change in *Lgr5* gene expression at hyperplasia as compared to other tumorigenic stages and also ~ 20-fold greater *Lgr5* expression level as compared to MMTV-PyMT hyperplasic foci. No major differences denoted in either *Lgr4* or *Lgr6* across tumorigenic stages and BC subtypes. (*n* = 1 with 3 replicates for WT, all C3(1)-Tag, and healthy MMTV-PyMT; *n* = 2 MMTV-PyMT for *Lgr4, Lgr6*, and *Lgr5* carcinoma, n = 4 MMTV-PyMT for *Lgr5* hyperplasia and adenoma) **b** Tamoxifen (Tam)-induced Cre recombinase knocked into the Lgr5 locus, *Lgr5*-EGFP-IRES-creERT2 crossed to Tomato reporter mice, Ai14(R26R dtTomato) indicated Lgr5^+^ cell progeny in hyperplasias (whole mounts, 1-week Tam treatment, 3 times/week) of the C3(1) Tag mouse model of TNBC. **c** Tumor volumes during treatment of LGR5-expressing TNBC PDX model mice over 4-weeks with anti-LGR5 (hu8E11v2)-MC-vc-PAB-MMAE (anti-LGR5-ADC, *n* = 8) and anti-gD-MC-vc-PAB-MMAE (Control *n* = 6). Arrow indicate administration of antibodies. **d** Tumor volumes of control mice and mice 4 weeks post treatment with anti-LGR5-ADC shows statistically significant decrease in overall tumor volume following inhibition of LGR5. Mean ± SD. (Student *t-*test, **P < 0.01)

Our DCIS TMA-derived data suggested a role for LGR5 in tumor initiation of BC with less favorable outcome. Gene expression analysis for *Lgr5* across various stages of tumorigenesis in *C3-(1) Tag* ER^−^ and *MMTV-PyMT* ER^+^ mouse models showed that *C3-(1) Tag* mice had upregulated expression of *Lgr5* at the hyperplastic stage whereas *MMTV-PyMT* showed no increase (Fig. 5a). Thus, using the *C3(1) Tag* ER^−^ BC mouse model, which develops primary tumors following BC progression characteristic of human BC [44, 50], we sought to further investigate the tumor-initiating capacity of *Lgr5*^+^ cells. To determine whether *Lgr5*^*+*^ cells possess tumor-initiating cells and whether they can generate progeny within hyperplastic lesions, we utilized tamoxifen-induced lineage-tracing in triple-transgenic C3(1) Tag;Lgr5-EGFP;Rosa^Tomato^ mice (Fig. 5b). Intriguingly, lineage tracing demonstrated that *Lgr5*^+^ cell progeny generated the bulk of hyperplastic mammary tumors (Fig. 5b). All hyperplastic foci within the tamoxifen-induced triple-transgenic mice exhibited red fluorescence, denoting the tumor-initiating capacity of *Lgr5*^*+*^ cells. Taken together, these data suggest a role for *Lgr5*^+^ cells as tumor-initiating cells in ER^−^ BC.

Given that LGR5 appears to have an important role in tumor initiation and progression of ER^−^ BC, we explored the ability to target LGR5 therapeutically. To recapitulate human BC, we took advantage of an ER^−^ BC PDX model. The PDX model is an attractive preclinical model to evaluate novel therapeutic alternatives since it harbors the malignant characteristics of tumors excised from BC patients, such as aberrations in copy number variations, epithelial-to-mesenchymal genes, and ER, PR, and HER2 expression [55]. We orthotopically transplanted the LGR5-expressing ER^−^ BC PDX line HCI-001 in NOD/SCID mice (Additional Fig. 3b) as previously described [38, 49]. Once tumors reached 150–200 mm^3^, we treated the mice with anti-LGR5 (hu8E11v2)-MC-vc-PAB-MMAE, a well-characterized antibody-drug conjugate (ADC) targeting LGR5 with minimal side-effects in mice such as minor weight loss [56]. Following 2 weeks of treatment, anti-LGR5-ADC treated tumors stopped growing and then significantly reduced in size while the control tumors continued to grow (Fig. 5c-d).

**Fig. 6:**
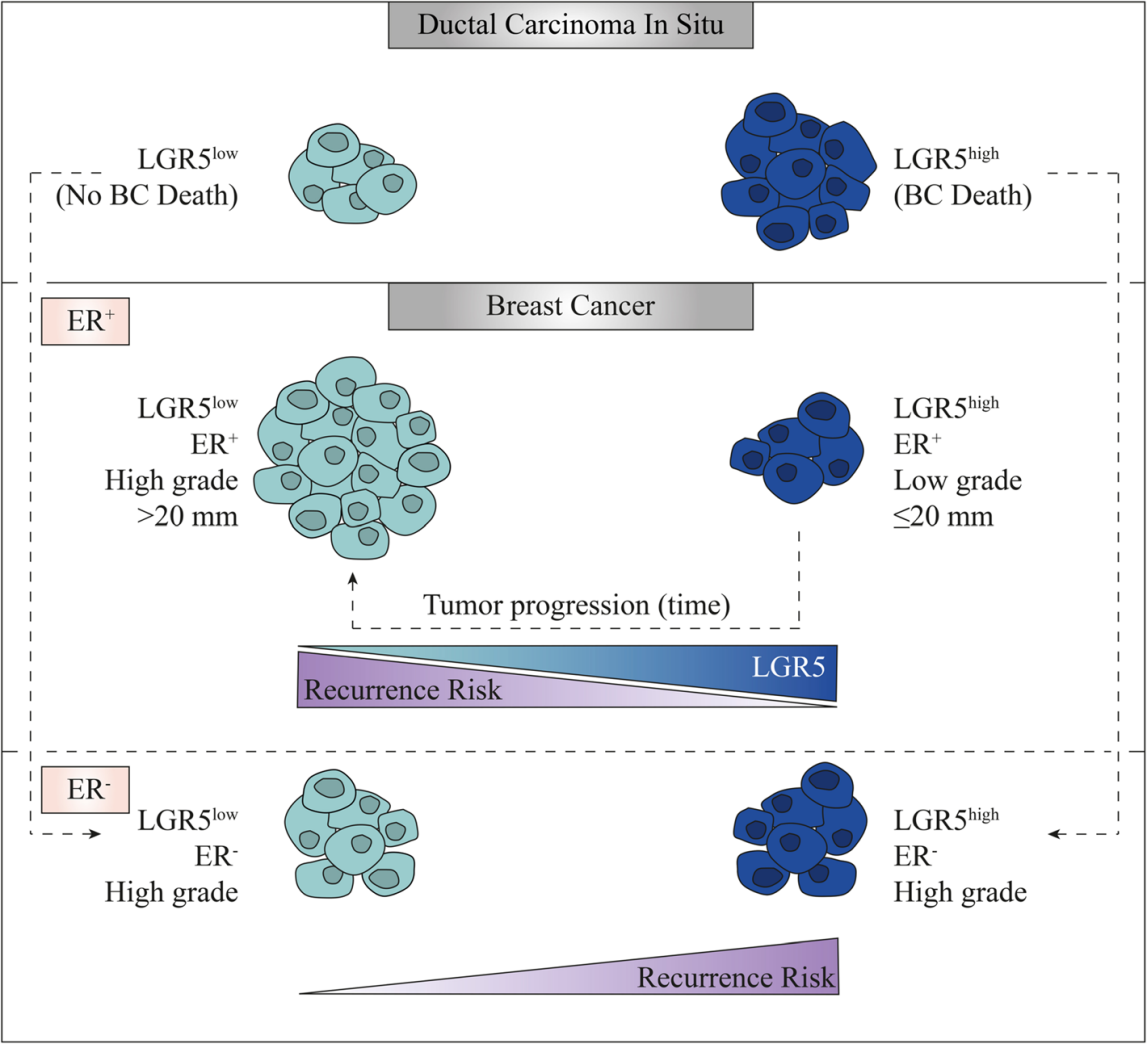
Schematic illustration of the prognostic value of LGR5 in ER^−^ vs. ER^+^ BC. Both LGR5^low^ and LGR5^high^ DCIS tumors can progress into BC, however only LGR5^high^ DCIS tumors progress into invasive tumors (possibly high-grade LGR5^high^ ER^−^) with related BC death. ER^+^ BC expressing high levels of LGR5 have a lower risk of recurrence, are smaller in size and of lower grade whereas ER^+^ BC expressing low levels of LGR5 have a higher risk of recurrence, are larger in size and of higher grade. It could be that as ER^+^ tumors progress, they become larger in size, of higher grade and lose their LGR5 expression. In stark contrast, high grade ER^−^ BC tumors with higher LGR5 expression stratifies a significantly greater recurrence rate as compared to tumors with lower LGR5 levels

In their totality, our data indicate the potential role of LGR5 both as a prognostic tool to determine malignant DCIS lesions alongside the value of utilizing LGR5 to stratify ER^+^ and ER^−^ BC tumor grade and recurrence (Fig. 6). The interrogation of clinical samples alongside in vitro and in vivo experiments in transgenic and PDX mouse models of BC demonstrates the potential of therapeutic intervention for TNBC patients by specifically targeting the tumorigenic LGR5^+^ tumor initiating cells.

## Discussion

The value of this study is several fold: First, we demonstrate an important differential prognostic value for LGR5 in ER^+^ vs. ER^−^ BC. Next, we showed evidence that LGR5 can help discern DCIS lesions that will become metastatic from those that will not progress into a fatal disease. Lastly, we performed in vitro and in vivo studies in various mouse models of BC to further substantiate a role for LGR5 in tumor initiation and solidify LGR5 as a valid therapeutic target for TNBC.

To date, the clinical importance of LGR5 for BC remains largely unexplored outside of two clinical studies that have correlated LGR5 with TNBC [37] and with tumor size and lymph node metastasis [57]. However, these studies did not determine the prognostic impact of LGR5 expression in ER^+^ vs. ER^−^ tumors separately. We have done so in the study described here, offering an explanation as to why some previous data appear contradictory to data presented here. In the study by Yang et al. the majority of patients (62%) had ER^−^ tumors [37], while clinically approximately 80% of all BC tumors are ER^+^ with considerably better outcomes compared to ER^−^ BC [6, 58]. In our BC TMA, 85% of the patients had ER^+^ tumors and our data pointed to distinct roles for LGR5 in ER^+^ vs. ER^−^ BC, presenting as a favorable variable for ER^+^ patients but unfavorable for high grade ER^−^ patients. The differential findings regarding the manifold roles of LGR5 in ER^+^ vs. ER^−^ BC might be partly explained by proliferative, estrogen-responsive Lgr5^+^ mammary cells [59] and a subset of dormant Lgr5^+^ Tetraspanin8 high (Tspan8^hi^) MaSC responsive to ovarian hormones [60]. Lgr5^+^Tspan8^hi^ MaSC, which are more quiescent as compared to Lgr5^+^Tspan8^−^ MaSC, may give rise to tumors that are distinct from those arising from the Lgr5^+^Tspan8^−^ MaSC pool with regards to ER status.

Furthermore, prevailing data indicate that BC tumors originate from non-invasive tumors, i.e., in situ carcinomas [61]. While not all in situ carcinoma tumors will progress into invasive tumors and not all invasive tumors will gain metastatic ability, some DCIS will gain the ability for invasion and metastatic advancement. In this study, our DCIS TMA-derived data illustrate the value of utilizing LGR5 as a prognostic marker for DCIS tumors that have the potential to advance into fatal BC. Notably, all of the DCIS patients included in our cohort (independent of ER status) who later died due to BC had LGR5^1–3^ DCIS tumors, while none of the patients with LGR5^0^ succumbed to BC. Hence, LGR5 expression may specifically associate with DCIS tumors that have the potential to advance into fatal BC (Fig. 6). Future studies with a larger cohort of DCIS patients are needed to further validate our findings and the prognostic impact of LGR5 for DCIS patients. While we did not determine the ER status of the recurred BC tumors from the DCIS patients, it would be valuable to know whether the patients with ER^+^ DCIS tumors, who later died due to BC, had ER^+^ or ER^−^ BC. Although ER^−^ DCIS never progress into ER^+^ BC, ER^+^ DCIS tumors can give rise to both ER^+^ and ER^−^ BC [62]. Our data could suggest that this may be partly attributed to the high expression of LGR5 and its association with DCIS tumors that will later progress into fatal high-grade ER^−^ BC.

Patients with high-grade ER^−^ BC generally have a poor prognosis with increased risk of recurrence and metastatic advancement. Here we show that LGR5 successfully differentiate patients within this BC subgroup with significantly lower risk of recurrence and death due to BC i.e. patients with LGR5^low^ tumors. This furthermore supports a role for LGR5, not only in tumor-initiation, but also for metastatic progression. Indeed, the critically important clinical issue in BC, as in many cancers, is metastasis. These are initiated by specialized cancer cells that bear a tumor-initiating capacity. To establish LGR5 as a valuable therapeutic target, we assessed and substantiated a role for LGR5^+^ cells in tumor initiation. In vivo studies performed by other groups and confirmed by us, have shown that LGR5^+^ ER^−^ BC cells bear an enhanced tumor-initiating capacity [37, 38]. In particular, we have done extensive work to prove the regenerative capabilities of LGR5^+^ cells [33]. Here we further substantiated a role for LGR5 in tumor initiation in TNBC by utilizing a lineage-tracing experiment in an autochthonous mouse model with high expression of LGR5 in hyperplastic ER^−^ TNBC. Based on this finding, we further revealed a therapeutic potential of anti-LGR5-ADC to target LGR5^+^ cells in aggressive ER^−^ BC. We showed that tumor growth is significantly attenuated upon administration of anti-LGR5-ADC using a human-in-mouse PDX model expressing elevated levels of LGR5. The utilization of anti-LGR5-ADC to target a smaller subset of potentially quiescent, tumorigenic LGR5^+^ tumor-initiating cells, which presumably are responsible for recurrence and metastatic progression [38, 63], presents a novel line of therapy for high-grade ER^−^ LGR5^high^ BC. Anti-LGR5-ADC therapy can help enhance the therapeutic potential of conventional chemotherapy that targets the bulk of proliferating BC cells [39]. Anti-LGR5-ADC therapy is also a logical combination with the newly approved Tecentriq-Abraxane combo, which enhances CD8^+^ T cell killing through inhibiting PD-L1 [27], to heighten complete tumor regression along with durable responses in the deadly high-grade ER^−^ LGR5^high^ BC. Beyond LGR5’s value as a prognostic marker, in a therapeutic scenario LGR5 can be utilized as a potential patient stratification tool.

## Conclusions

We showed that LGR5 expression could potentially assist in preventing overtreatment by distinguishing indolent DCIS tumors from those that might progress into lethal BC. LGR5 expression can also be used to recognize high-grade ER^−^ BC tumors with increased risk of recurrence and death due to BC, a prognostic criterion that would enhance clinical BC diagnosis. Furthermore, we demonstrated proof-of-concept feasibility in using anti-LGR5-ADC as a novel therapeutic alternative for TNBC patients that could potentially be combined with currently approved checkpoint inhibitor combination for TNBC to promote durable complete response. Taken together, our data illustrates the clinical potential of using LGR5 as a diagnostic and prognostic biomarker as well as a therapeutic target in high grade ER^−^ BC.

## Supplementary information

**Additional file(s): Figure 1.** Prognostic role of LGR5 in BC. Kaplan-Meier plots (log-rank test) displaying recurrence-free survival according to LGR5 levels in BC TMA. **A.** Not-grouped and **b.** Dichotomized into LGR5^low (0–1)^ and LGR5^high (2–3)^ tumor staining intensity. Kaplan-Meier plot (log-rank test) displaying **c.** recurrence free survival, and **d.** death due to BC, in low-grade ER^−^ BC according to LGR5 dichotomized into LGR5^low (0–1)^ and LGR5^int/high (2–3)^ tumor staining intensity. **Figure 2.** Prognostic role of LGR5 in DCIS according to ER status. Kaplan-Meier plots (log-rank test) displaying time to death due to BC according to LGR5 dichotomized into LGR5^low (0)^ and LGR5^int/high (1–3)^ tumor staining intensity in **a**. ER^−^ DCIS and **b**. ER^+^ DCIS. **c.** Distribution of No recurrences, In situ or Invasive recurrence in LGR5-DCIS subgroups (numbers 0,1–3 indicate tumor staining intensity). **Figure 3.** Gene expression analysis of *Lgr4*, *Lgr5*, and *Lgr6.***a**. Quantitative polymerase chain reaction on human BC cell lines examining *Lgr4, 5, and 6* gene expression levels. Efficient knockdown of LGR5 alongside higher levels of canonical LGR5 in TNBC as compared to Luminal A BC. **b.** Analysis of publicly available microarray data of PDX models (accession number GSE32531) identifies *Lgr5* as highly expressed in HCI-001 xenograft 5th generation. HCI-001 xenograft 5th generation was used in transplant experiments within NOD/SCID mice followed with anti-LGR5-ADC treatment. **Table 1.** Cox regression analyses for recurrence free survival in women with ER+ primary breast cancer. **Table 2.** Cox regression analyses for recurrence free survival (RFS) in women with high-grade ER- primary breast cancer. **Table 3.** Cox regression analyses for breast cancer specific survival (BCSS) in women with high-grade ER- primary breast cancer.

## Data Availability

The datasets used and/or analysed during the current study are available from the corresponding author(s) on reasonable request.

## Abbreviations

DCIS: Ductal carcinoma in situ
ER: Estrogen receptor
BC: Breast cancer
HER2: Human epidermal growth factor receptor 2
TNBC: Triple negative breast cancer
PR: Progesterone receptor
LGR5: Leucine-rich repeat containing G-protein-coupled receptor 5
MaSC: Mammary stem cell
CSC: Cancer stem cells
TMA: Tissue microarray
PDX: Patient-derived xenograft
ADC: Antibody drug conjugate (ADC)
KD: Knockdown
IHC: Immunohistochemistry
rtPCR: Reverse transcription polymerase chain reaction
