# Supplementary material for: LGR5 in breast cancer and ductal carcinoma in situ: a diagnostic and prognostic biomarker and a therapeutic target
Source: BMC Cancer. 2020 Jun 10;20:542. doi: 10.1186/s12885-020-06986-z (PMC7285764; doi:10.1186/s12885-020-06986-z)
Supplement: Supplementary file 1 — Additional file(s): Figure 1. Prognostic role of LGR5 in BC. Kaplan-Meier plots (log-rank test) displaying recurrence-free survival according to LGR5 levels in BC TMA. A. Not-grouped and b. Dichotomized into LGR5low (0–1) and LGR5high (2–3) tumor staining intensity. Kaplan-Meier plot (log-rank test) displaying c. recurrence free survival, and d. death due to BC, in low-grade ER− BC according to LGR5 dichotomized into LGR5low (0–1) and LGR5int/high (2–3) tumor staining intensity. Figure 2. Prognostic role of LGR5 in DCIS according to ER status. Kaplan-Meier plots (log-rank test) displaying time to death due to BC according to LGR5 dichotomized into LGR5low (0) and LGR5int/high (1–3) tumor staining intensity in a. ER− DCIS and b. ER+ DCIS. c. Distribution of No recurrences, In situ or Invasive recurrence in LGR5-DCIS subgroups (numbers 0,1–3 indicate tumor staining intensity). Figure 3. Gene expression analysis of Lgr4, Lgr5, and Lgr6.a. Quantitative polymerase chain reaction on human BC cell lines examining Lgr4, 5, and 6 gene expression levels. Efficient knockdown of LGR5 alongside higher levels of canonical LGR5 in TNBC as compared to Luminal A BC. b. Analysis of publicly available microarray data of PDX models (accession number GSE32531) identifies Lgr5 as highly expressed in HCI-001 xenograft 5th generation. HCI-001 xenograft 5th generation was used in transplant experiments within NOD/SCID mice followed with anti-LGR5-ADC treatment. Table 1. Cox regression analyses for recurrence free survival in women with ER+ primary breast cancer. Table 2. Cox regression analyses for recurrence free survival (RFS) in women with high-grade ER- primary breast cancer. Table 3. Cox regression analyses for breast cancer specific survival (BCSS) in women with high-grade ER- primary breast cancer. [file 12885_2020_6986_MOESM1_ESM.pdf]

Additional Figure 1

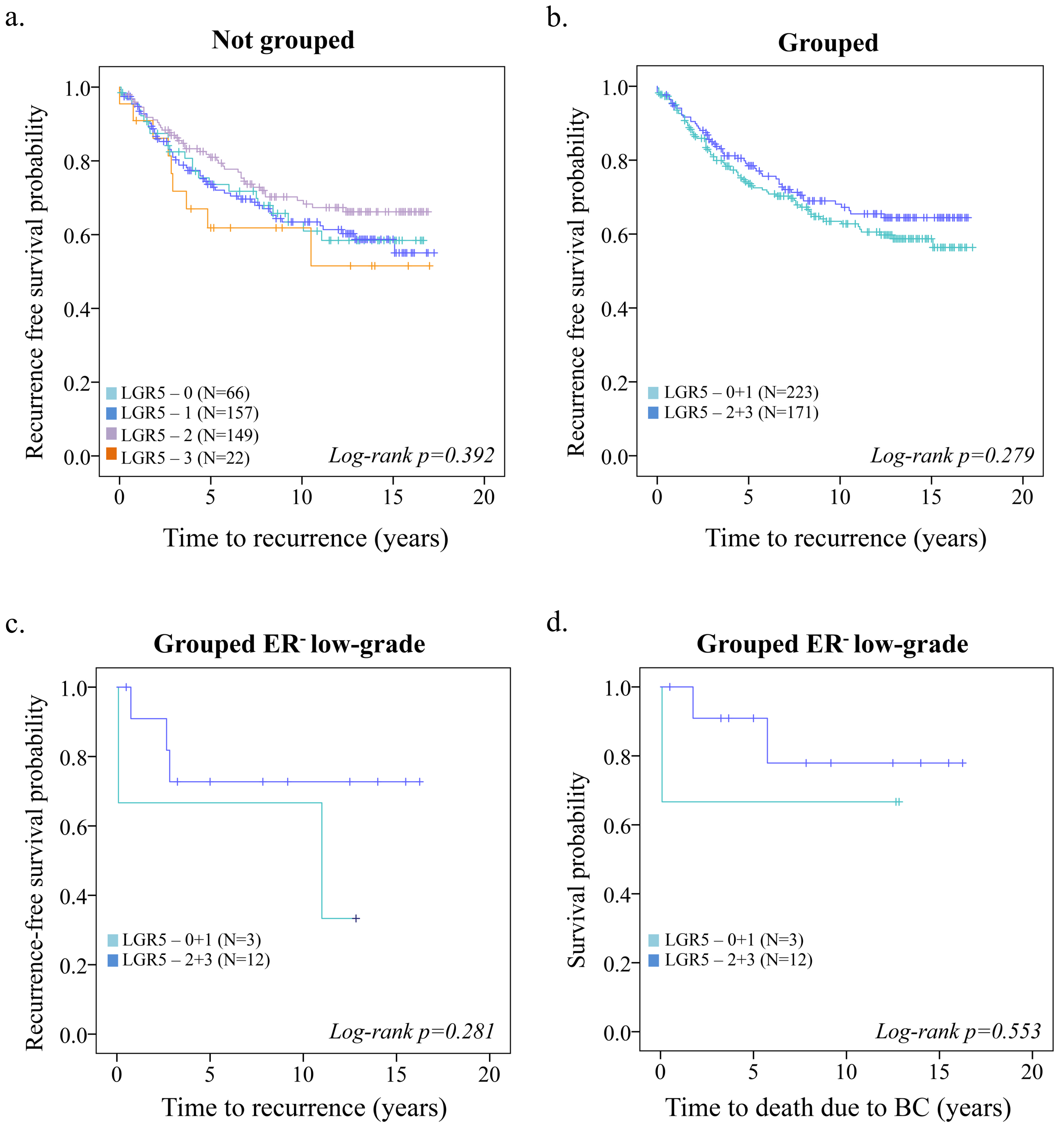

Additional Figure 2

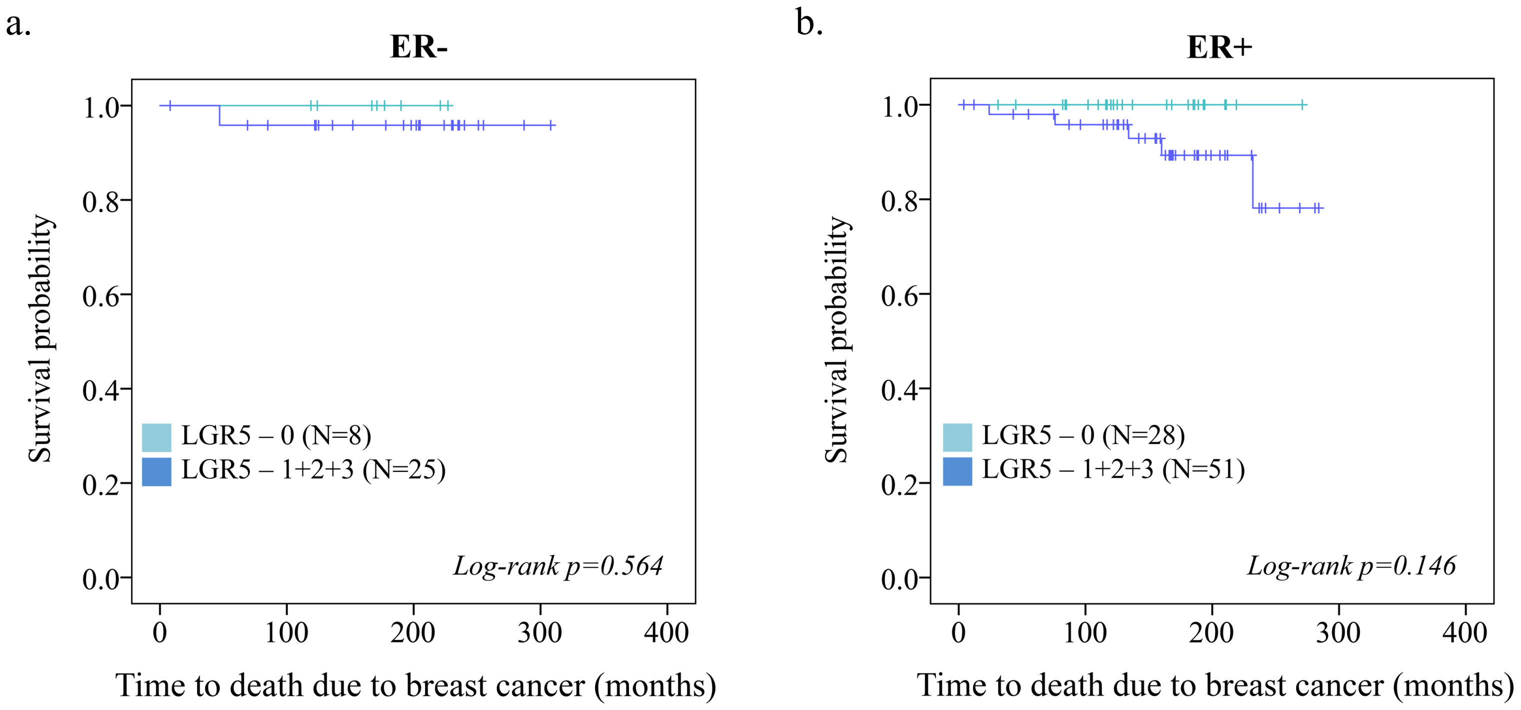

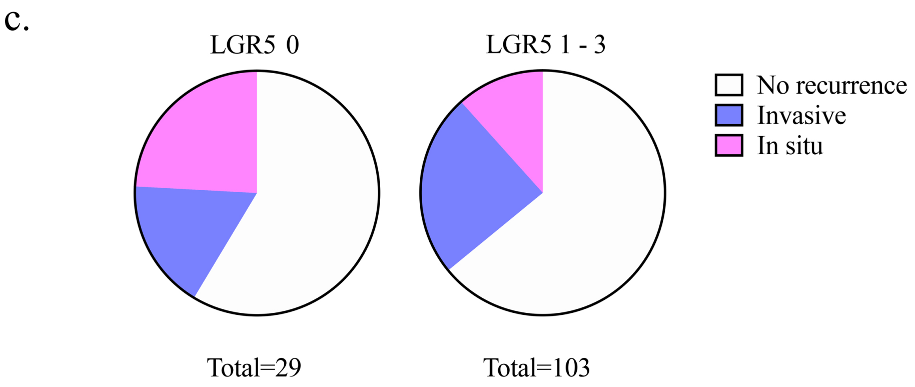

Additional Figure 3

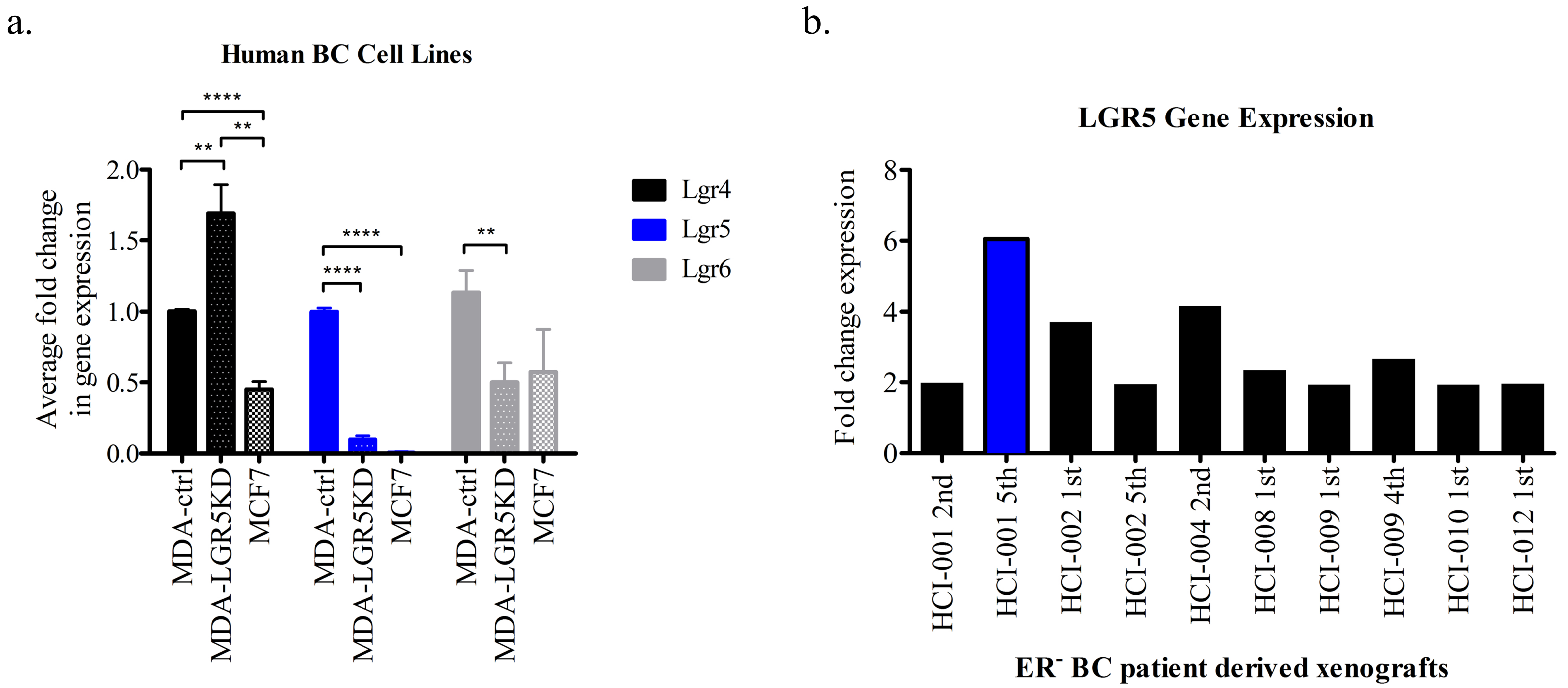

Additional Table 1 Cox regression analyses for recurrence free survival in women with ER+ primary breast cancer

**Variable**	**HR**	**(95% CI)**	***P* value**	**HR**	**(95% CI)**	***P* value**
	Univariable			Multivariable		
Tumor size						
≤20 vs 20	2.16	(1.50-3.11)	**0.000**	1.18	(0.77-1.82)	0.45
Nodal metastasis						
- vs +	3.33	(2.28-4.89)	**0.000**	2.36	(1.53-3.62)	**0.000**
NHG						
1, 2 vs 3	3.30	(2.78-4.60)	**0.000**	2.69	(1.78-4.06	**0.000**
LGR5						
low vs high	0.64	(0.43-0.95)	**0.028**	0.75	(0.49-1.15)	0.18

Abbreviations: HR, hazard ratio; NHG, Nottingham histologic score

LGR5, Leucine-rich repeat-containing G-protein coupled receptor 5. Bold indicates P-value <0.05

Additional Table 2 Cox regression analyses for recurrence free survival (RFS)
in women with high-grade ER- primary breast cancer

**Variable**	**HR**	**(95% CI)**	***P* value**	**HR**	**(95% CI)**	***P* value**
	Univariable			Multivariable		
Tumor size						
≤20 vs 20	1.54	(0.66-3.58)	0.311	2.26	(0.77-6.65)	0.14
Nodal metastasis						
- vs +	2.50	(1.04-6.04)	**0.041**	2.91	(1.07-7.88)	**0.036**
LGR5						
low vs high	3.36	(1.29-8.77)	**0.013**	2.64	(0.94-7.43)	0.065

Abbreviations: HR, hazard ratio; CI, confidence intervals; NHG, Nottingham histologic score
LGR5, Leucine-rich repeat-containing G-protein coupled receptor 5. Bold indicates P-value <0.05

Additional Table 3 Cox regression analyses for breast cancer specific survival (BCSS) in women with high-grade ER- primary breast cancer

**Variable**	**HR**	**(95% CI)**	***P* value**	**HR**	**(95% CI)**	***P* value**
	Univariable			Multivariable		
Tumor size						
≤20 vs 20	2.12	(0.79-5.69)	0.134	3.21	(0.84-12.29)	0.088
Nodal metastasis						
- vs +	4.20	(1.48-11.94)	**0.007**	4.71	1.38-16.18	**0.014**
LGR5						
low vs high	4.03	(1.29-12.55)	**0.016**	3.08	(0.89-10.60)	0.075

Abbreviations: HR, hazard ratio; CI, confidence intervals; NHG, Nottingham histologic score

LGR5, Leucine-rich repeat-containing G-protein coupled receptor 5. Bold indicates P-value <0.05
